# SynchroSep-MS: Parallel LC Separations for Multiplexed Proteomics

**DOI:** 10.1021/jasms.5c00207

**Published:** 2025-07-30

**Authors:** Noah M. Lancaster, Li-Yu Chen, Bingnan Zhao, Benton J. Anderson, Mitchell D. Probasco, Vadim Demichev, Daniel A. Polasky, Alexey I. Nesvizhskii, Katherine A. Overmyer, Scott T. Quarmby, Joshua J. Coon

**Affiliations:** † Department of Chemistry, 5228 University of Wisconsin-Madison, Madison, Wisconsin 53706, United States; ‡ Department of Biomolecular Chemistry, University of Wisconsin-Madison, Madison, Wisconsin 53706, United States; § 145254 Morgridge Institute for Research, Madison, Wisconsin 53715, United States; ∥ Quantitative Proteomics Laboratory, 14903 Charité − Universitätsmedizin Berlin, Berlin 10117, Germany; ⊥ Department of Pathology, 1259 University of Michigan, Ann Arbor, Michigan 48109, United States; # Department of Computational Medicine and Bioinformatics, University of Michigan, Ann Arbor, Michigan 48109, United States; ¶ National Center for Quantitative Biology of Complex Systems, Madison, Wisconsin 53706, United States

## Abstract

Achieving high throughput remains a challenge in MS-based proteomics for large-scale applications. We introduce SynchroSep-MS, a novel method for parallelized, label-free proteome analysis that leverages the rapid acquisition speed of modern mass spectrometers. This approach employs multiple liquid chromatography columns, each with an independent sample, simultaneously introduced into a single mass spectrometer inlet. A precisely controlled retention time offset between sample injections creates distinct elution profiles, facilitating unambiguous analyte assignment. We modified the DIA-NN workflow to effectively process these unique parallelized data, accounting for retention time offsets. Using a dual-column setup with mouse brain peptides, SynchroSep-MS detected approximately 16,700 unique protein groups, nearly doubling the peptide information obtained from a conventional single proteome analysis. The method demonstrated excellent precision and reproducibility (median protein %RSDs less than 4%) and high quantitative linearity (median R^2^ greater than 0.96) with minimal matrix interference. SynchroSep-MS represents a new paradigm for data collection and the first example of label-free multiplexed proteome analysis via parallel LC separations, offering a direct strategy to accelerate throughput for demanding applications such as large-scale clinical cohorts and single-cell analyses without compromising peak capacity or causing ionization suppression.

## Graphical abstract

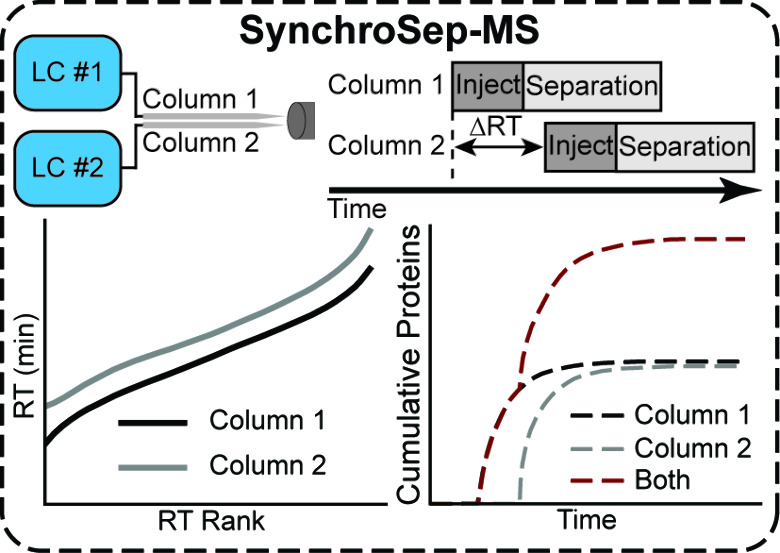

## Introduction

Advances in mass spectrometry (MS)-based proteomics are largely driven by improvements in instrumentation, including more comprehensive and high-throughput proteomics methods; however, obtaining complete human proteomes has typically required extensive fractionation, multiple injections, and long analysis times.^1,2^ Recent advances in instrumentation that allow for MS/MS acquisition rates exceeding 200 Hz, i.e., Astral mass analyzer, have greatly accelerated the rate and depth of proteome analysis. Specifically, these high MS/MS acquisition rates can be combined with data-independent acquisition (DIA) to enable detection of over 10,000 proteins from human samples in just tens of minutes.^3–11^ With one sample at a time, two or three samples per hour can now be analyzed with excellent proteomic depth. That said, many clinical and single cell applications, for example, demand the analysis of hundreds and even thousands of samples.

To satiate this throughput demand, many have now focused on the chromatographic separation step. Indeed, active effort in the field seeks to dramatically accelerate the speed of chromatographic analysis through short gradients,^5,12–19^ or parallelization of sample loading/column washing with gradient separations using multiple columns.^20–33^ Shortening gradients, however, both erodes quantitative precision and accuracy and reduces proteomic depth. Multiplexing can be achieved through isotopic labeling, e.g., tandem mass tags, but that approach has its own complications, including cost, considerably more preparation labor, ratio distortion, and incompatibility with DIA.

We supposed a novel route to multiplexed, label-free DIA proteome analysis could be achieved by use of multiple LC columns, each with their own sample and integrated nanoelectrospray emitter, which could be analyzed in parallel owing to the speed of the new Orbitrap Astral MS platform. This approach would eliminate the loss of ionization efficiency that occurs when multiple samples are loaded onto a single column and would provide the higher ion flux needed so that the MS system could operate at top speeds. Here we describe an initial implementation of this approach, which we call SynchroSep-MS.^34^

## Experimental Section

### Materials and Reagents

Ultrapure water was provided by a Barnstead GenPure Pro system (Thermo Scientific). Trifluoroacetic acid (TFA, HPLC grade, >99.9%), chloroacetamide (≥98%, C0267–100G), urea (U5378–1kg), tris­(2-carboxyethyl)­phosphine hydrochloride (TCEP, C4706–2G, guanidine hydrochloride solution (6M, SRE0066), and fetal bovine serum (F2442) were obtained from Sigma-Aldrich. Formic acid (LC-MS grade), methanol (Optima LC/MS grade), acetonitrile (Optima LC-MS grade), Tris Buffer (1 M Tris pH 8.0, 0.2 μm filtered, Invitrogen, AM9856), and Pierce Peptide Retention Time Calibration Mixture (PRTC, 88320) were obtained from Fisher Scientific. Lysyl Endopeptidase (LysC, 100369–826) was sourced from VWR. Sequencing grade Modified Trypsin (V5113) was obtained from Promega. BCA Protein Assay Reagent A (23228), BCA Protein Assay B (23224), Bovin Serum Albumin Standards (2 mg/mL, 23209), IMDM (12440053), and penicillin–streptomycin (15140122) were obtained from Thermo Scientific.

### Sample Preparation

All experiments were performed in accordance with the National Institutes of Health Guide for the Care and Use of Laboratory Animals and were approved by the Animal Care and Use Committee at the University of Wisconsin-Madison. Brains were harvested from C57BL/6J adult female mice after euthanasia and immediately frozen in liquid nitrogen. The brains were pulverized under liquid nitrogen into a fine powder.

Mouse brain samples were weighed on dry ice, and guanidine buffer (5.4 M guanidine hydrochloride, 100 mM Tris, pH 8) was added. Tissue lysis was performed via vortexing and sonication in a chilled sonicator bath. The protein concentration was measured using a Pierce BCA protein assay kit.

Human HAP1 KO cells acquired from Horizon Discovery were cultured in IMDM with 1% penicillin–streptomycin and 10% fetal bovine serum at 37 °C and 5% CO_2_, as previously described.^11,35^ Lysis was performed in 6 M guanidine with sonication. The protein concentration was then measured via a Pierce BCA protein assay kit.

After measuring the protein content of samples via the BCA assay, methanol was added to 90% to precipitate proteins and the samples were centrifuged at 9000g for 5 min. The supernatant was removed, and the protein pellets were resolubilized in 8 M urea, 100 mM Tris, 10 mM TCEP, and 40 mM chloroacetamide, pH 8 at a target protein concentration of 1.5 mg/mL. The samples were diluted to 2 M urea with 100 mM Tris, pH 8. LysC was added at a 1:50 enzyme:protein ratio, and the samples were rocked gently for 4 h at ambient conditions. Trypsin was then added at a 1:50 enzyme:protein ratio, and the samples were rocked gently overnight at ambient conditions. Digestion was quenched with 10% TFA in water, and the samples were centrifuged for 5 min at 9000g prior to desalting with Strata-X 33 μm polymeric reversed phase SPE cartridges (Phenomenex). Desalted samples were dried down in a SpeedVac (Thermo Scientific) and reconstituted in 0.2% formic acid in water. The peptide concentration was measured via a NanoDrop (Thermo Scientific) prior to LC-MS/MS analysis.

### Data Collection

To implement a dual column SynchroSep-MS setup, two nanoLCs were used. A Vanquish Neo UHPLC (Thermo Scientific) was configured on the mass spectrometer control computer. A contact closure line connected the Vanquish Neo to an Ultimate 3000 nLC (Thermo Scientific) such that the Vanquish Neo could send an output signal that was then received by the Ultimate 3000. The Ultimate 3000 was configured and controlled by a separate computer. Sample injection onto Column 1 was performed using the Vanquish Neo nLC. At the end of the sample injection step for the Vanquish Neo, a relay output signal was programmed into the method. The Ultimate 3000 method was programmed to start sample loading only after it received a relay signal from Vanquish Neo. Thus, the retention time offset could be consistently maintained. By adjustment of the time delay of the relay signal in the Vanquish Neo method, the absolute retention time offset can be tuned.

For both nLCs, mobile phase A was 0.2% formic acid in water, and mobile phase B was 80% ACN/20% water/0.2% formic acid. Columns were prepared by laser pulling emitters on silica capillaries (360 μm O.D., 75 μm I.D.) and packing to 40 cm with 1.7 μm BEH C18 particles, 130 Å (Waters) at ultrahigh pressure using a custom-built high pressure packing station.^36^ For the Vanquish Neo, a gradient separation was performed at 0.3 μL/min starting by ramping from 0 to 6% B over 2 min, then ramping from 6 to 47%B for 30 min, washing at 100% B for ∼11.5 min, and equilibrating at 0% B for ∼25 min. Note that this equilibration stage was extended to ensure mass spectrometry data collection was performed throughout the entire gradient separation on the second column and could likely be reduced to maximize throughput. A relay signal was programmed into the Vanquish Neo method at 0, 1, or 2 min for the retention time offset tuning data set. For most of this study, the relay signal was sent at the 1 min time point in the Vanquish Neo method. For Ultimate 3000, sample injection was delayed until the relay signal was detected as an input. Upon the relay signal, sample injection was performed by drawing up 8 μL of transfer fluid (0.2% formic acid in water), followed by drawing up the sample and 2.4 μL of transfer fluid using a 5 μL sample loop. The injection valve was then switched to inject onto the column. A gradient was ramped from 0 to 7% B over 2 min, followed by ramping from 7% B to 43% B over 30 min. Then, the %B was set to 100%B over 0.1 min followed by ∼11.7 min of washing, and 10 min of equilibration at 0% B. The gradient conditions are slightly different for each LC as the specific settings were tuned to provide well distributed total ion chromatograms for the two columns. For this method, reproducible timing of injections is critical to maintaining consistent retention time offsets. In our experience, the Vanquish Neo system exhibits higher variability in the total sample loading times, likely due to the injection preparation steps and sample loop pressurization step implemented. While this variability would not negatively impact typical performance since the retention time start is synchronized across separations, the use of a Vanquish Neo to control the second column might lead to variation in the delay between injections onto the first and second columns. We thus chose to employ an Ultimate 3000 to perform separations, as this system does not perform as many injection preparation steps or sample loop pressurization and takes consistent amounts of time for sample loading across injections.

Both emitters were aligned to the inlet capillary using a 3D-printed holder. A spray voltage of 2000 V was applied to both columns via a liquid–liquid junction. The ion transfer tube temperature was set to 280 °C. A DIA method was employed. Here, 240,000 resolving power MS1 scans were collected in the Orbitrap analyzer with an *m*/*z* range of 380–980, with RF Lens (%) of 40, maximum injection time of 5 ms, and normalized AGC target of 500%. Astral MS2 scans were acquired with a DIA method iterating over a DIA *m*/*z* range of 380–980 with 4 Th windows (with 1 Th overlap). The scan range in the Astral analyzer was from 150 to 2000 *m*/*z*, and the normalized AGC target was set to 500% with a maximum injection time of 5 ms and an RF Lens (%) of 40. The cycle time for MS1 scans was set to 0.6 s.

### Data analysis

Raw data files were converted to the.mzml format with MSConvert (v. 3.0.241192ef69)^37^ using the peakPicking filter set to ‘Vendor’. Dual injection files were searched against a mouse protein database using the default settings for the ‘DIA_SpecLib_Quant’ workflow within FragPipe.^38,39^ The intermediate output files *{file-name}_*rank­{*rank number*}.pepXML files were parsed using Python, where rank denotes the ranks of PSM quality as implemented in the MSFragger-DIA algorithm. Peptide Spectral Matches (PSMs) in .pepXML files were filtered for an expectation value less than 1e-5. Duplicate PSMs were filtered for only pairs with a retention time difference greater than 30 s. The earlier PSM from each pair was assumed to be from Column 1 and the later from Column 2. The RTs from each column were used to construct a locally weighted scatterplot smoothing (LOWESS) model with respect to the Column 1 RTs using the ‘lowess’ function within the Statsmodels library^40^ and the ‘interp1d’ function within SciPy.^41^ The ΔRT model was defined as the difference between these models. The Pierce PRTC standard raw data were manually examined in Skyline (v. 24.1.0.414) to define experimental ΔRT values.^42^

A prototype version of DIA-NN 2.2.0 was employed for searching the data.^43^ The prototype version takes in two additional parameters through the command line, --rt-shift and --rt-gap. The --rt-shift parameter defines the retention time offset (ΔRT) that will be applied to the retention times in the spectral library for searching a specific file. The --rt-gap parameter specifies the retention time window width that is used to search the chromatogram around the specified retention time. A Column 1-only injection is used for creating a retention time-aligned predicted spectral library by searching in DIA-NN and aligning retention times using an R script. A workflow diagram for the data processing procedure is shown in . For the dilution series experiments, normalization was turned off using the --no-norm command. For data analysis, the following columns in the report parquet output by DIA-NN were filtered for values less than or equal to 0.01: Q.Value, Lib.Q.Value, Global.Q.Value, Lib.PG.Q.Value, Global.PG.Q.Value, PG.Q.Value, Lib.Peptidoform.Q.Value and Global.Peptidoform.Q.Value.

Normal searches were performed by using a mouse protein database downloaded from UniProt. The entrapment search was performed by combining a mouse and *C. elegans* protein database downloaded from UniProt.^44^ Match-between-runs (MBR) and protein inference were turned off for a precursor-level FDR assessment. The ‘Q.Value’ column was filtered for values ≤ 0.01. For the entrapment search, the false discovery proportion (%) was calculated according to

$$\mathrm{FDP}\left(\%\right)=\frac{N_{\varepsilon }}{N_{\tau }+N_{\varepsilon }}\times 100$$

Here, N_ε_ represents the number of *Caenorhabditis elegans* entrapment precursors, and N_τ_ represents the number of mouse target precursors. Precursors corresponding to both *C. elegans* and mouse protein identifiers were considered to be targets.

Figures were created in Python 3.11.5 using the matplotlib library.^45^ Pearson correlations and linear regressions were calculated using ‘pearsonr’ and ‘linregress’ within SciPy.^41^ The code used for data analysis and visualization is available at https://github.com/coongroup/SynchroSep-MS. Raw data and search results have been uploaded to MassIVE and are available at accession number MSV000098329. A description of the files provided is available in .

## Results and Discussion

To explore the feasibility of SynchroSep-MS we constructed two capillary LC columns and aligned their integrated emitters using a 3D-printed holder (Figure 1A and B), placing them both in front of the atmospheric pressure inlet of an Orbitrap Astral MS system. Each column was connected to a nanoLC pump, such that loading and gradient elution were controlled independently (Figure 1C). To distinguish which column/sample an analyte originates from, we developed an injection scheme (Figure 1D) that imparts a time delay between the two separations such that any given analyte present in both samples will be detected by the MS system across two elution profiles separated by a defined temporal gap (Figure 1E and F).

**Figure 1:**
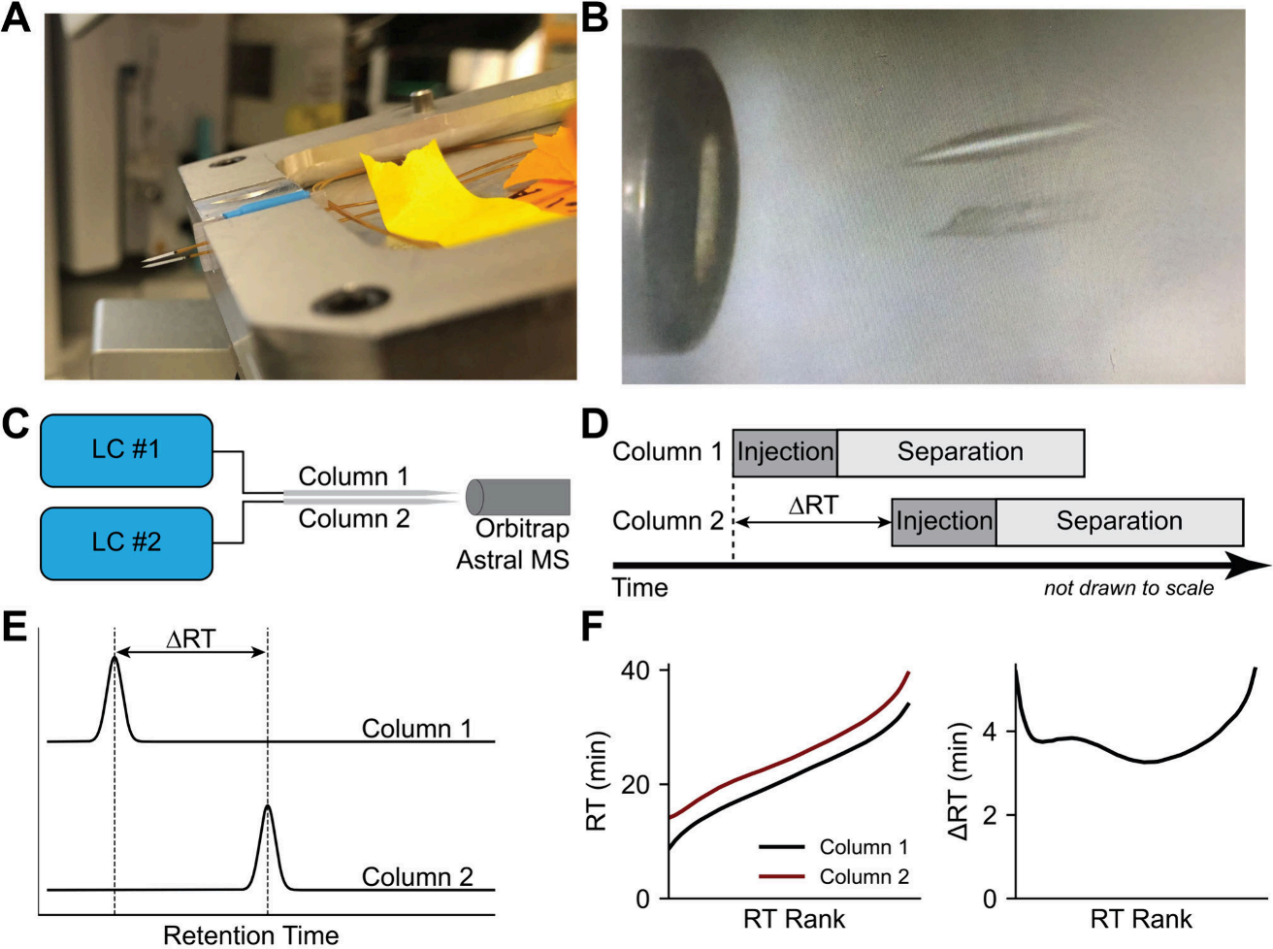
SynchroSep-MS conceptual overview. (A) Two packed capillary columns (75 μm I.D.) with integrated emitters installed in a custom-built column heater with a 3D-printed alignment piece. (B) Side view of emitters aligned to the MS inlet. (C) Two (or more) LCs are connected to two (or more) columns and the emitters are aligned to the mass spectrometer inlet. (D) An injection onto the first column is performed, and a gradient separation is begun, followed by injecting a sample onto the second column and beginning a second gradient separation. The delay between sample injections can be controlled, allowing an adjustment of the resulting retention time offset (ΔRT). (E) The resulting data will have duplicate peaks in the chromatograms with a retention time offset of ΔRT, representing peaks from Column 1 and Column 2. (F) The retention time offset (ΔRT) can be modeled to determine the ΔRT throughout the gradient, which varies slightly throughout the gradient due to experimental variations in the columns and LC conditions.

Figure 2 presents initial results using the SynchroSep-MS technique to analyze tryptic peptides derived from mouse brain protein isolates. Here, identical samples were injected onto each column and analyzed using a 4 min time delay and a DIA method iterating over *m*/*z* 380–980 with 4 *m*/*z* bins (Orbitrap Astral). For comparison, three chromatograms are shown: – (Figure 2A) peptides loaded only on Column 1 and blank on Column 2, (Figure 2B) peptides loaded only on Column 2 and blank on Column 1, and (Figure 2C) peptides loaded on both columns. From Figure 2C, we observe that the injection of peptides on both columns produces a chromatogram that appears as the superposition of the two single column chromatograms. Note the dual column injection resulted in a total ion intensity that is 94% of the sum of both single column injections. To determine how consistent the introduced retention time offset was across all peptides, we used the preliminary PSM files (.pepXML) generated by the MSFragger-DIA workflow^39^ in FragPipe to construct a model of the retention time offset (ΔRT). Specifically, duplicated PSMs were used to calculate the retention time offset throughout the separation, and then, a local LOWESS regression model was fit to the data (Figure 2D). From these data we conclude that the retention time offset was centered around 4 min; however, it varies throughout the chromatogram likely due to experimental differences between chromatographic setups. That said, this variation was small relative to the absolute delay, and this delay can be tuned experimentally (). To further validate this conclusion, we injected a cocktail of 15 synthetic peptides onto both columns and repeated the experiment. Manual inspection of these results shows excellent agreement with the local regression model (Figure 2E).

**Figure 2:**
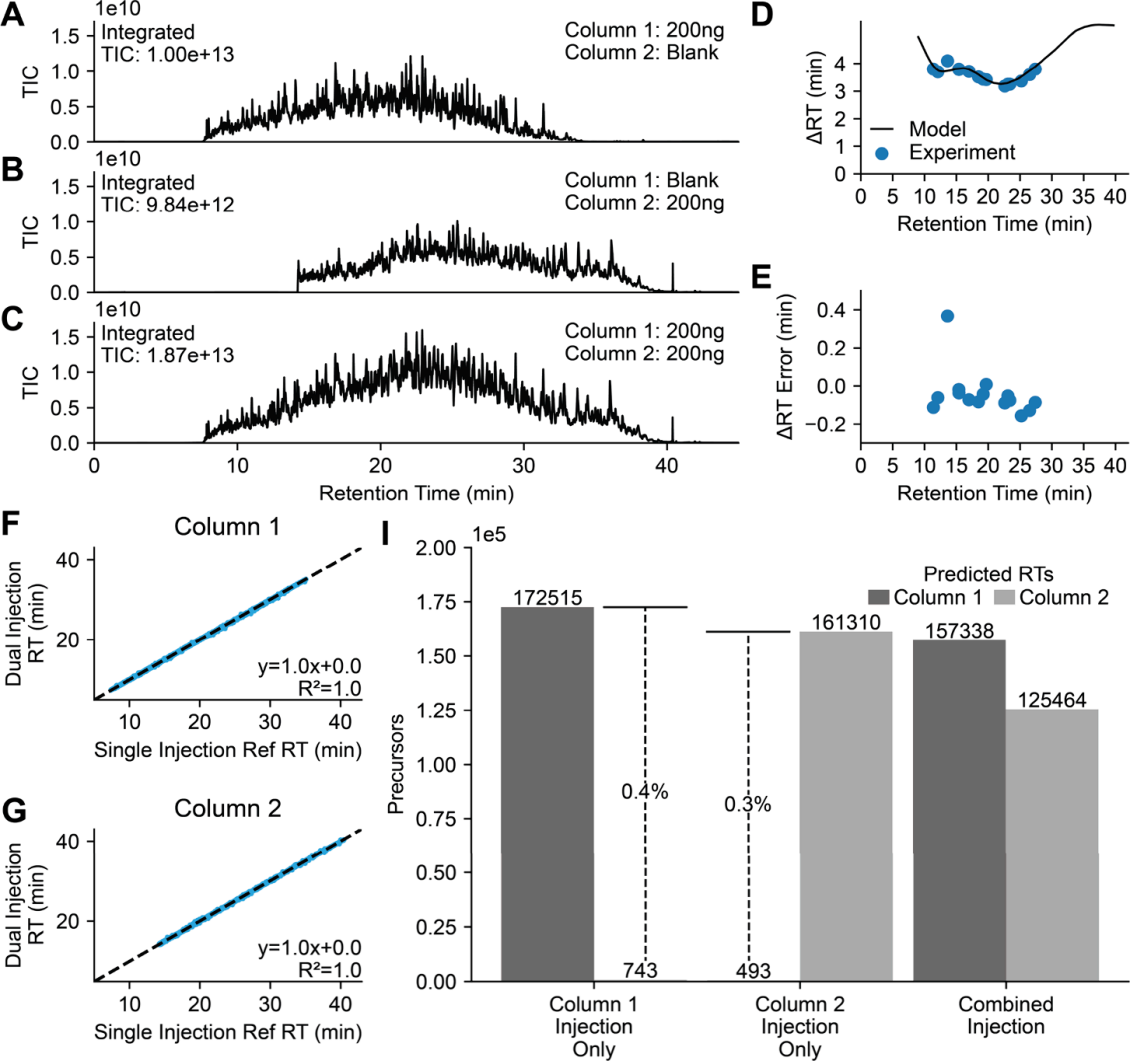
Assignment of peaks to the originating column. (A) Representative chromatograms for 200 ng mouse brain peptide loads onto (A) Column 1-only, (B) Column 2-only, and (C) both columns at once are shown. Injections onto both columns result in a chromatogram that is essentially the sum of the individual injection chromatograms. (D) LOWESS model of the retention time offset, ΔRT, is created based off of PSMs observed twice in a FragPipe-DIA search. Experimental ΔRT values observed for synthetic peptide standards (Pierce PRTC standard mix) are plotted as blue dots. The experimental ΔRT values are based off the observed retention time differences between PRTC peaks across 6 observations (3 single column injections each, 3 dual column injections). The LOWESS model was created from the data shown in (C). (E) The error in the ΔRT model is calculated based on the experimental values. Here, the model is accurate to ± 0.4 min, which is small relative to the ∼4 min ΔRT shown in (B). Retention times of precursors detected after searching the data shown in (A), (B), and (C) using the prototype DIA-NN software were plotted against each other for (F) Column 1 (172,515 total overlapping precursors used for the analysis) and (G) Column 2 (161,310 total overlapping precursors used for the analysis). Strong agreement between the RTs detected in the single-column injections and dual-column injections (as indicated by a linear regression slope of 1, y-intercept of 0, and R^2^ of 1.0) indicate accurate peak assignments. (I) Single column injections for the chromatograms shown in (A) and (B) were searched against both the Column 1 and Column 2 predicted RTs, alongside the dual column injection in (C). False positive rates of 0.4% and 0.3% were observed for columns 1 and 2, respectively, indicating accurate column assignment.

From these data, we conclude that the SynchroSep-MS concept is viable, and for full implementation, we next required an informatic workflow for global data processing. Accordingly, we modified the DIA-NN software to allow analysis of these results based on a retention time-aligned predicted library and the time offset (see Experimental Section and for workflow details). Using the mouse brain peptide results described above, we validated the modified software’s ability to accurately assign peptide precursors to the column from which it eluted. Figure 2F and 2G plot the retention times of the precursors identified in the single and dual injection data. The strong agreement confirms that the software is correctly assigning peptides to the originating column. Figure 2I shows the number of identified precursors assigned to each column across the three files. For example, when peptides are injected only on Column 1 (Figure 2A), 172,515 precursors were assigned to Column 1, while only 743 were assigned to Column 2. These 743 are, of course, false positives and constitute only 0.4% of the total precursors detected. Similar results were obtained when peptides were injected on Column 2 (Figure 2B). When samples were loaded onto both columns, we detected the vast majority of peptides from the single column injections (91% and 78%). Validation of the retention time window width for processing is demonstrated in . We also performed an entrapment search of single- and dual-injection data on both columns against a combined *C. elegans*/Mouse protein database and observed consistent false discovery proportions across the different data sets, providing confidence that FDR control is not negatively impacted for this new type of data ().

Figure 3A presents the number of proteins detected in the three experiments discussed above. To summarize: Column 1-only (∼9,000), Column 2-only (∼8,700), and Column 1 and 2 (i.e., SynchroSep, ∼ 8,700 from Column 1 and ∼ 8,000 from Column 2 for a total of ∼ 16,700). A more intuitive visual of these results is shown in a plot of proteins detected over time (Figure 3B) and here we see the additive nature of SynchroSep-MS and how nearly identical protein depth for each sample can be achieved with parallel analysis. The precursor-level results are shown in . As expected, the protein identifications lost in the dual injections relative to the single injections correspond to lower intensity proteins (). Next, we examined the quantitative reproducibility of the method. Figure 3C shows excellent precision for quantitation with median RSDs < 5% across >7000 protein groups (see for zoom-in). Analysis at the precursor ion level shows similar trends (). These data confirm our guiding supposition that fast-scanning MS/MS technology is outpacing our relatively static approach to one LC column and on the sample at a time.

**Figure 3:**
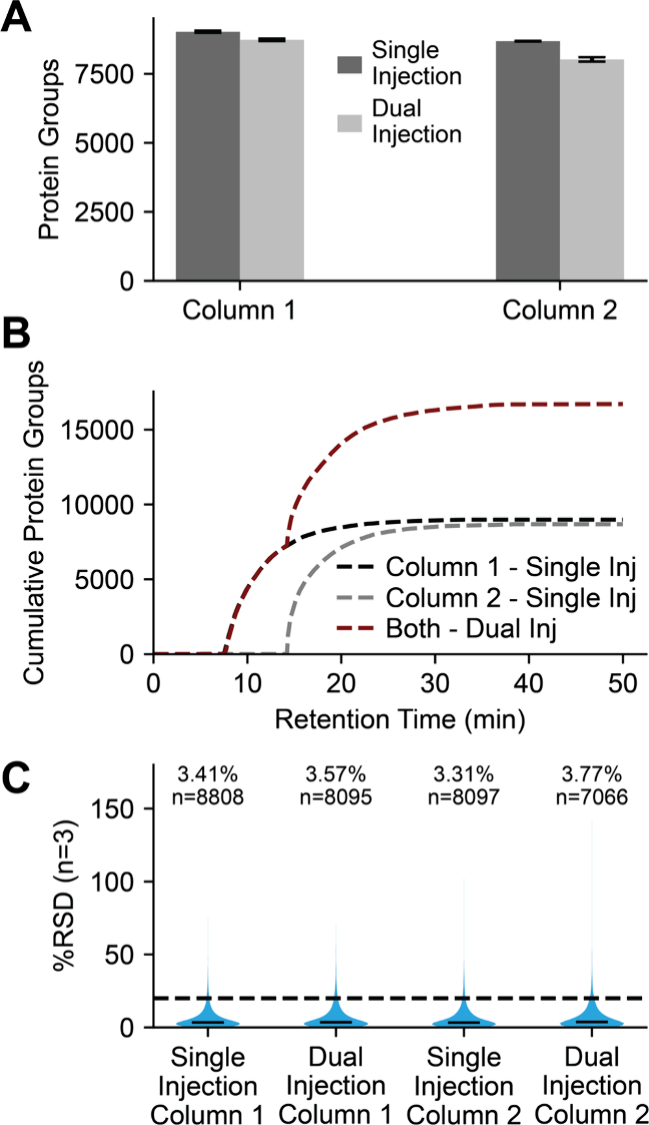
Protein group identifications. (A) Number of protein groups detected on Column 1 and Column 2 for a 200-ng mouse peptide load as either a single injection (with blank injected on the other column) or a dual column injection (200 ng mouse peptide load on the other column) are shown. Error bars represent the minimum and maximum values observed across triplicate injections. (B) The cumulative protein group identifications as a function of retention time are shown for a 200-ng load only on Column 1, a 200-ng load on Column 2, and 200-ng loads on both columns. For the injection on both columns, protein groups assigned to different columns are treated as unique identifications. (C) Protein groups detected across triplicate injections were used to calculate %RSD values for quantification for the dual- and single-injections onto Columns 1 and Column 2. The median %RSD and number of protein groups included in each data set are shown above each violin plot. The dashed line indicates %RSD = 20.

One interesting observation in this data set was that the depth achieved for Column 2 was more severely affected by the dual-column injection than Column 1 (Figure 3A). In this experimental setup, elution from Column 1 begins prior to and ends before the elution of peptides from Column 2. Thus, during the first 5–6 min of elution from Column 1, there is no notable signal from Column 2 (Figure 2A–C). Because most precursor identifications are generated earlier in the gradient (), the identification performance of Column 1 might uniquely benefit from the absence of signal from Column 2 that would increase spectral complexity (and decrease the likelihood of identifications due to the potential decreased dynamic range, signal-to-noise, etc.). In contrast, the identification rate for peptides eluting later from Column 2 might benefit from the lack of interference from Column 1 at the end of the gradient, although the lower rate of precursor identification observed at the end of the gradient for the single-column injections () suggests that this might not compensate for the loss of identifications earlier in the gradient. To further investigate this phenomenon, we binned the precursors identified in the single-injection data by retention time and calculated the proportion of these precursors that were missing in the dual-injection identifications (). With this analysis, we observe that Column 2 has a higher proportion of missing precursor identifications in the earlier part of the gradient, while Column 2 has a higher proportion of missing precursors in the later part of the gradient. Additionally, the magnitude of the trend seems to be stronger for Column 2 as suggested by the data in . This aspect of our SynchroSep-MS data set helps to explain why Column 2 demonstrates a worse performance loss for dual-injections than Column 1 and suggests that increased spectral complexity caused by the addition of more columns for a higher degree of multiplexing would further diminish the identification performance. However, we hypothesize that the continued development of faster and more sensitive mass spectrometers will improve the capacity to handle complex mixtures and decrease the magnitude of this phenomena.

Ultimately, SynchroSep-MS provides an ideal and novel avenue for multiplexed and high-throughput proteome analysis. To achieve this goal, the approach must also provide reliable quantification. Accordingly, we performed two dilution series experiments in triplicate. The first reduced the amount of peptides loaded on each column equally across a range (Direct) and the second evaluated the effect of interference between columns by varying the loading amounts in opposite directions (Inverted) (Table 1).

**Table 1:** Mouse Peptide Dilution Series Scheme

Direct Dilution Series		Inverted Dilution Series	
Column 1	Column 2	Column 1	Column 2
10 ng	10 ng	200 ng	10 ng
50 ng	50 ng	100 ng	50 ng
100 ng	100 ng	50 ng	100 ng
200 ng	200 ng	10 ng	200 ng

The Direct dilution series results are as expected – that is, as the load level is reduced the protein identifications also drop (Figure 4A, see for precursor level results). Importantly, similar protein identification trends are observed in the Inverted dilution series, except that they move in opposing directions (Figure 4B, see for precursor level results). We note that in both experiments, Column 1 outperforms Column 2, which is likely due to variation in separation quality and/or emitter positioning. To evaluate quantitative performance, we calculated R^2^ values across these data and plotted the distributions for each dilution series in Figure 4C. The median R^2^ value was over 0.95 for all experiments (and over ∼ 0.9 at the precursor level, ), suggesting excellent quantitative performance. Another performance requirement for SynchroSep-MS is that the measured quantities across the columns are consistent. We assessed this figure of merit by plotting the quantities of proteins detected across Columns 1 and 2 in the Direct Dilution Series against each other and observed excellent correlation (Figure 4D). A slope of 0.989 and y-intercept of 0.063 indicate that the quantities are nearly identical with Column 1 having slightly higher abundances. The same analysis at the precursor level yields a slope of 0.93, indicating a similar trend (). Similar results were observed when performing the same analysis for the Inverted Dilution series (). This difference can easily be corrected by normalization approaches and likely can be eliminated by optimization of the experimental setup (i.e., emitter positioning). We also compared the abundances measured across the two dilution series schemes on Column 1 as shown in Figure 4E. Strong agreement at the protein quantity level (and the precursor level, ) indicates that the method is able to selectively quantify analytes from both columns without notable interference.

**Figure 4:**
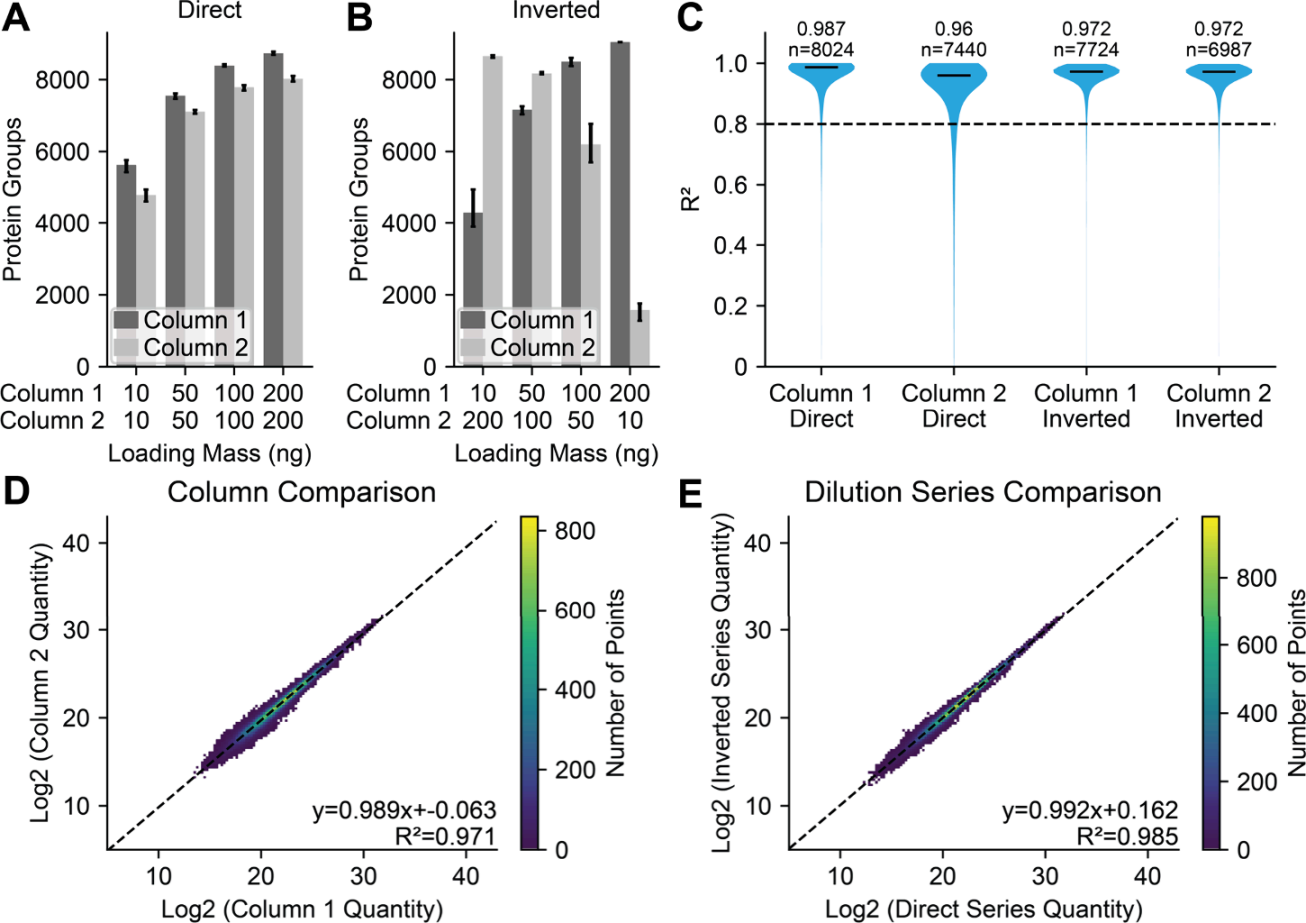
Performance of SynchroSep-MS method assessed with dilution series. The number of detected protein groups is shown for (A) the Direction Dilution Series and (B) the Inverted Dilution Series described in Table 1. The direct dilution series consists of injections of the same loading mass onto both columns, whereas the inverted dilution series uses different loads to represent an extreme case of a varied background matrix from the other column. Error bars represent the minimum and maximum values observed across triplicate injections. (C) The R^2^ value distributions for Column 1 and Column 2 across the dilution series shown in Table 1 are shown. Values were calculated only for protein groups detected across at least three concentration points. The median R^2^ value and number of protein groups included in each distribution are shown above each plot. The dashed line indicates an R^2^ value of 0.8. (D) The log2-transformed quantities for protein groups detected across both Columns 1 and 2 in the Direct Dilution series are plotted against each other. A slope less than 1 indicates that Column 1 protein quantities are generally higher. (E) The log2-transformed quantities for protein groups detected across both dilution series are plotted against each other. A slope less than 1 indicates that the direct dilution series protein quantities are generally higher. For this example, data on Column 1 is shown.

## Conclusions

Using SynchroSep-MS, we can simultaneously analyze two proteomes, acquiring nearly double the peptide information from a single proteome analysis, demonstrating that modern, fast-scanning mass analyzers are capable of handling the increased analysis complexity of this method. Our data also show high quantitative precision and reproducibility for analysis of a complex mammalian proteome. Further dilution series experiments demonstrate good quantitative linearity and suggest that minimal matrix interference is present for multiplexed analysis.

We conclude that the SynchroSep-MS method represents a new paradigm for data collection and is the first example of label-free multiplexed proteome analysis. Although this proof-of-concept work demonstrates the method using two columns, we envision a multicolumn setup with four or more columns that could significantly improve analysis throughput. This multiplexed setup would be especially attractive for large-scale clinical cohorts and single-cell analyses.

The main current limitation with large-scale implementation of SynchroSep-MS is the relative complexity of the LC pump setup. In our current efforts, each column has an independent pump. Moving forward, we envision splitting the flow of a single pump across the various columns while still maintaining the ability to load each independently. Elimination of these technical hurdles will allow a very direct path to accelerate the throughput of proteome analysis. Further, as MS analyzers continue to increase in acquisition speed, SynchroSep-MS offers a strategy to boost throughput without having to compress gradient lengths–which doubtlessly causes reduced peak capacity and ionization suppression of the low abundance analytes.

## Supplementary Material





## Notes

Processing SynchroSep-MS using Prototype DIA-NN software; tuning the Retention Time Offset; RT window width scouting for DIA-NN processing; external FDR assessment via entrapment analysis; precursor identifications; dual vs single-injection dynamic range; protein group RSD zoom in; RT binning analysis of dual column SynchroSep-MS data; performance of SynchroSep-MS method assessed with dilution series – precursor level; column Intensity comparisons for the inverted dilution series (PDF)
File map for available data (XLSX)
