# Supplementary material for: SynchroSep-MS: Parallel LC Separations for Multiplexed Proteomics
Source: J Am Soc Mass Spectrom. 2025 Jul 30;36(9):1979–87. doi: 10.1021/jasms.5c00207 (PMC12331150; doi:10.1021/jasms.5c00207)
Supplement: Supplementary file 1 [file js5c00207_si_001.pdf]

Supporting Information

SynchroSep-MS: Parallel LC Separations for Multiplexed Proteomics

Noah M. Lancaster[1,2], Li-Yu Chen[1,2], Bingnan Zhao[2], Benton J. Anderson[2], Mitchell D. Probasco[2,3], Vadim Demichev[4], Daniel A. Polasky[5], Alexey I. Nesvizhskii[5,6], Katherine A. Overmyer[2,3,7], Scott T. Quarmby[2,7], and Joshua J. Coon[1,2,3,7]*

[1] Department of Chemistry, University of Wisconsin-Madison, Madison, WI 53706, USA
[2] Department of Biomolecular Chemistry, University of Wisconsin-Madison, Madison, WI 53706, USA
[3] Morgridge Institute for Research, Madison, WI 53715, USA
[4] Quantitative Proteomics Laboratory, Charité – Universitätsmedizin Berlin, Berlin 10117, Germany
[5] Department of Pathology, University of Michigan, Ann Arbor, MI 48109, USA
[6] Department of Computational Medicine and Bioinformatics, University of Michigan, Ann Arbor, MI 48109, USA
[7] National Center for Quantitative Biology of Complex Systems, Madison 53706, WI
* Correspondence: J.J.C. (coon@wisc.edu)

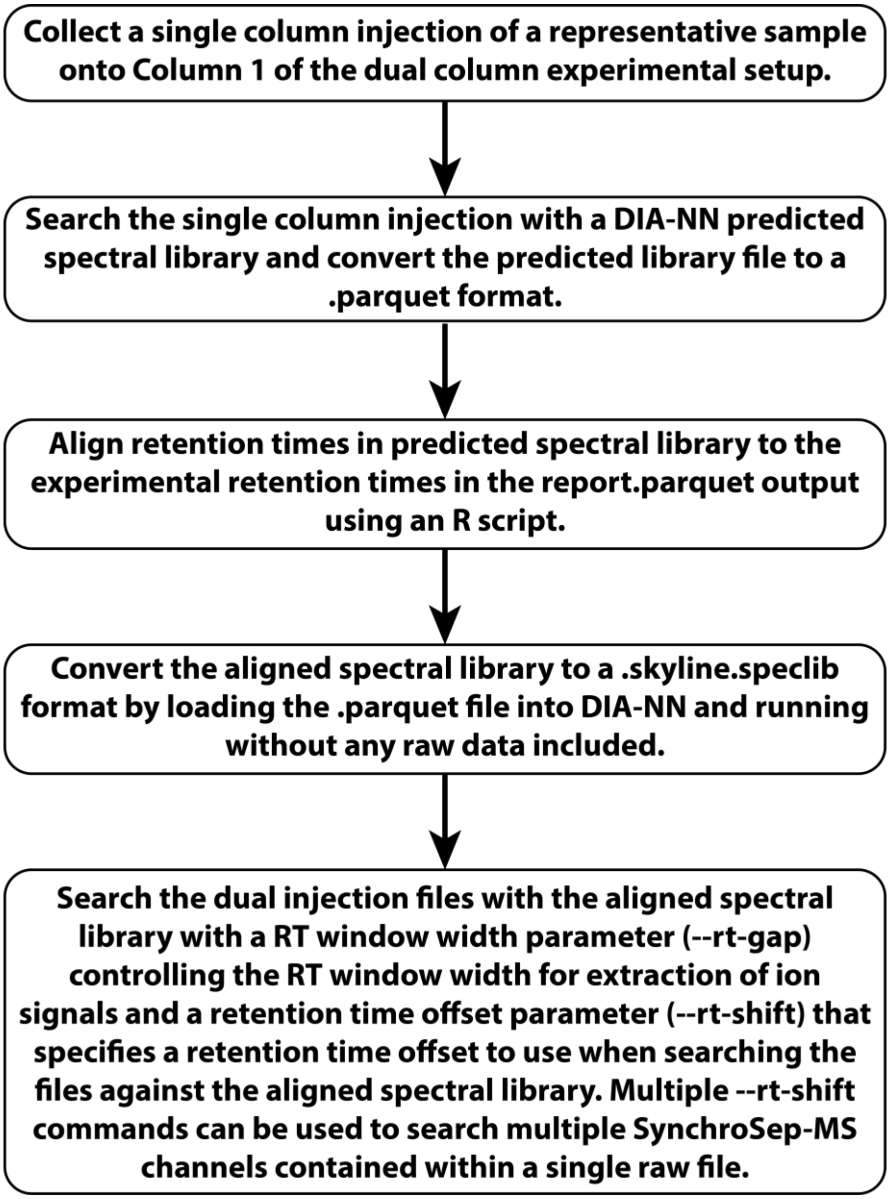

Figure S1: Processing SynchroSep-MS using Prototype DIA-NN Software. A schematic workflow is shown for processing the dual column SynchroSep-MS data with a modified version of the DIA-NN software.

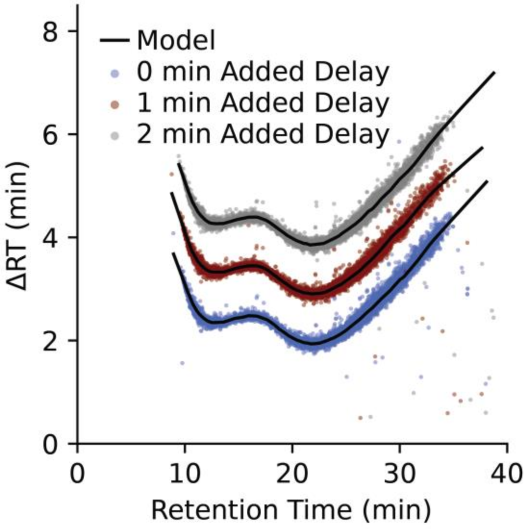

Figure S2: Tuning the Retention Time Offset. The ΔRT models for three analyses of a HAP1 digest with 0, 1 and 2 minutes added delay between the injections onto Column 1 and Column 2 are shown. Dots represent the ΔRT for features detected by MSFragger-DIA, and the lines represent the local regression model generated from these points. For this study, the '1 min Added Delay' method was selected as this method offered a balance between parallelization of the separations and a ΔRT magnitude large enough for confident column assignment. Note that the delay for the '0 min Added Delay' represents the minimum retention time offset achievable for this particular experimental setup, but this could be reduced by experimental adjustments.

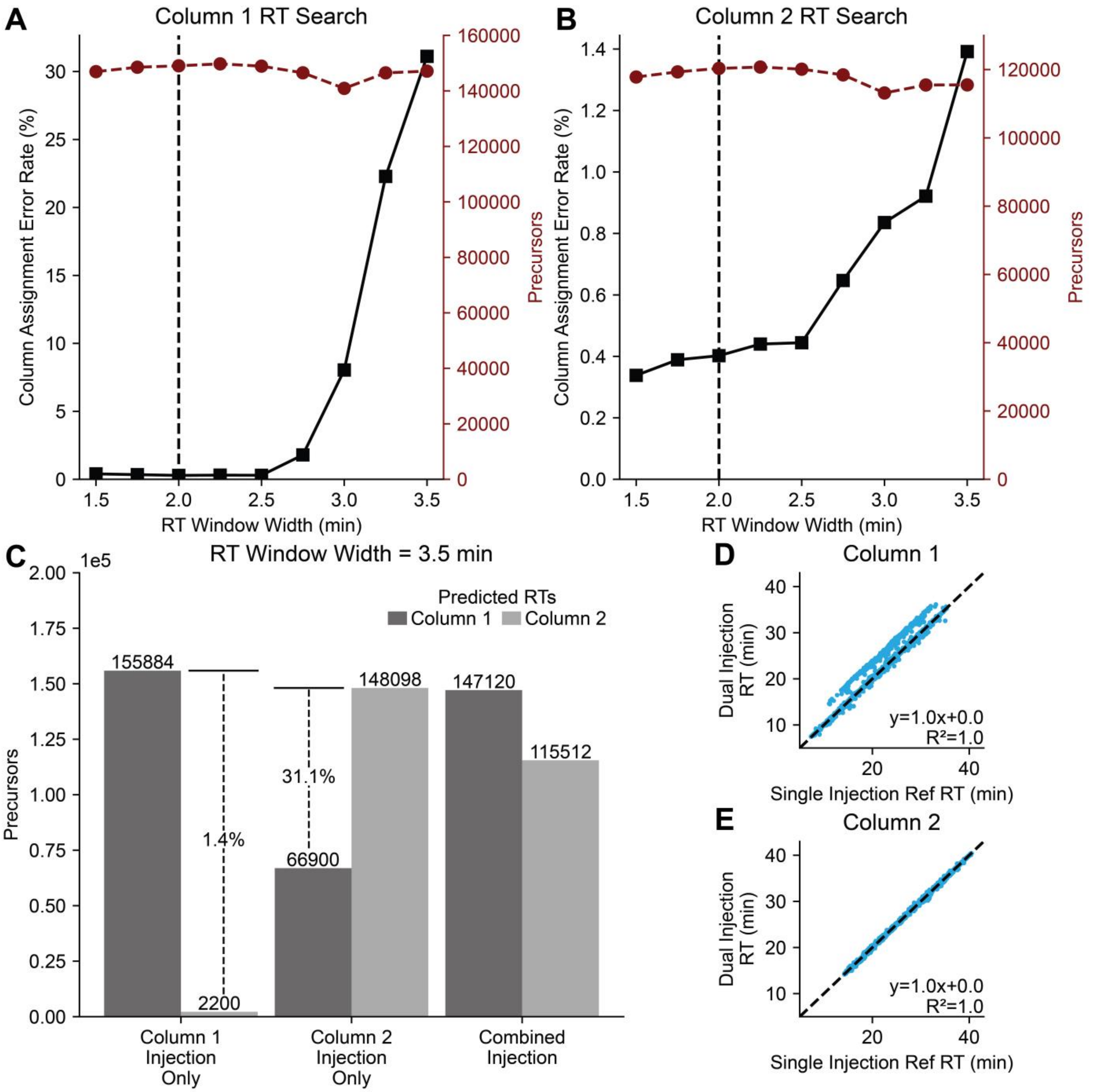

Figure S3: RT Window Width Scouting for DIA-NN Processing. The RT window width (which is controlled by the --rt-gap parameter in DIA-NN) dictates how widely around the predicted retention time DIA-NN should look when searching for peptide features. The error rate for assignments of precursors to an originating column can be assessed by searching data from a single-injection file with predicted retention times for both Column 1 and Column 2. For an injection of sample onto just Column 1 (with a blank on Column 2), any precursors detected with the predicted RTs for Column 2 would represent incorrect assignments. The error rate can then be calculated as the ratio of incorrect assignments/total assignments when using the predicted RTs for Column 1. Note that the reported results are for analysis with a predicted library (i.e. library-free) and we suppose that empirical DIA-based library (e.g. made from bulk samples and used on multi-column analysis of single cells) could result in improved assignment accuracy. The

calculated Column Assignment Error Rates (%) are shown for a Column 1-only injection (A) and a Column 2- only injection (B) alongside the total number of precursors detected when using the appropriate predicted retention times. The dashed line indicates the window width used for analysis in this study. As an example of what can go wrong if the RT window is too large, the results for a search using a 3.5-minute window width are shown in panels (C), (D), and (E). The single column injections for the chromatograms shown in **Figures 2A** and **2B** were searched against both the Column 1 and Column 2 predicted RTs, alongside the dual column injection in **Figure 2C**. False positive rates of 1.4% and 31.1% were observed for Column 1 and Column 2, respectively. The retention times of precursors detected for a dual-column and single-column injection for (E) Column 1 and (F) Column 2 are plotted against each other. Strong agreement between the RTs (as indicated by a linear regression slope of 1, y-intercept of 0, and R^2 of 1.0) in the single and dual column injections is observed for Column 2. However, the wider RT window leads to many precursors detected from Column 2 being assigned to Column 1, which is observed as a second cluster of precursors detected at an offset retention time relative to the reference retention time for the single column injection.

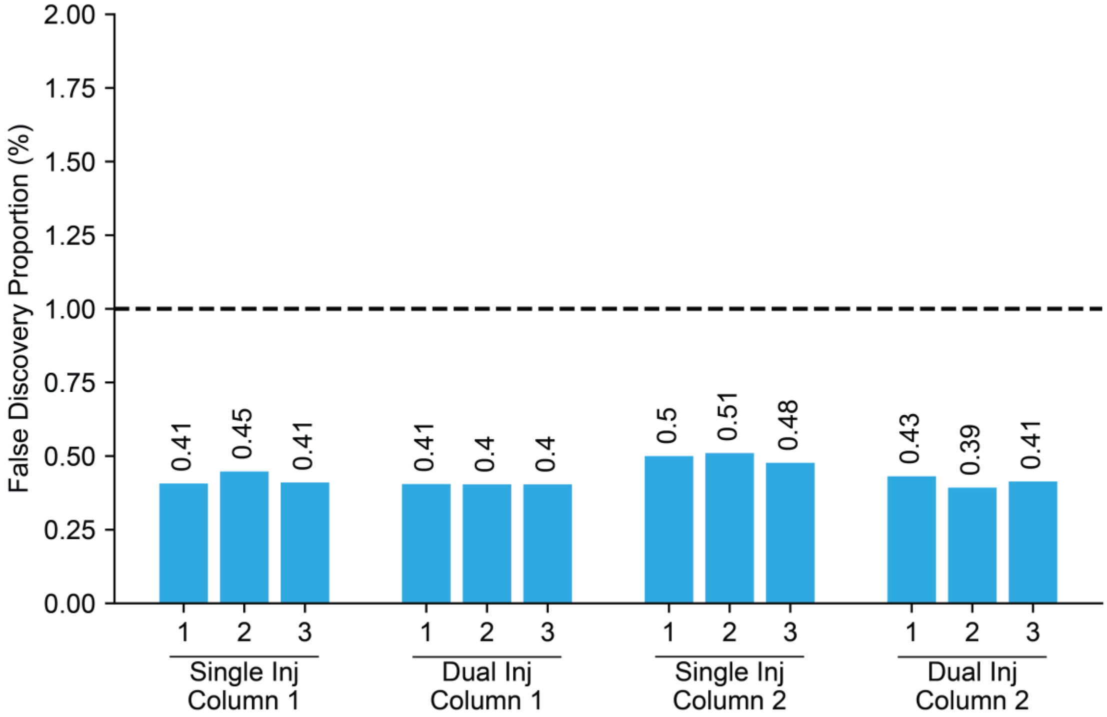

Figure S4: External FDR Assessment via Entrapment Analysis. The calculated false discovery proportion (%) values at the precursor level are shown for replicate injections on both columns. The dashed line indicates the 1% FDR level. 'Single Inj' refers to a datafile generated by mouse peptides injection onto only a single column with a blank loaded on the other. 'Dual Inj' refers to a datafile generated by injecting mouse peptides onto both columns. The entrapment search was performed using the workflow described in **Figure S1** with a combined *C. Elegans*/mouse protein database.

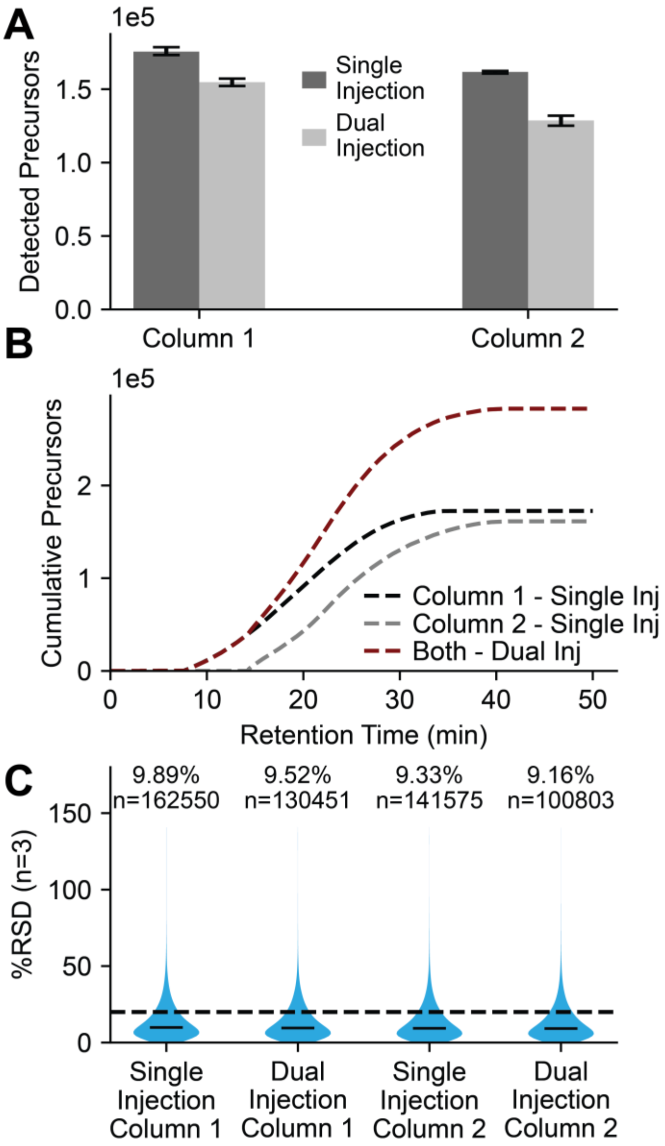

Figure S5: Precursor Identifications. (A) The number of precursors detected on Column 1 and Column 2 for a 200-ng mouse peptide load either as a single injection (with blank injected on the other column) or a dual column injection (200-ng mouse peptide load on the other column). Error bars represent the minimum/maximum values observed across triplicate injections. (B) The cumulative precursor identifications as a function of retention time are shown for a 200-ng load only on Column 1, a 200=ng load on Column 2, and 200-ng loads on both columns. For the injection on both columns, precursors assigned to different columns are treated as unique identifications. (C) Precursors detected across triplicate injections were used to calculate %RSD values for quantification for the dual- and single-injections onto Column 1 and Column 2. The median %RSD values and number of precursors included in each dataset are shown above each violin plot. The dashed line indicates %RSD = 20.

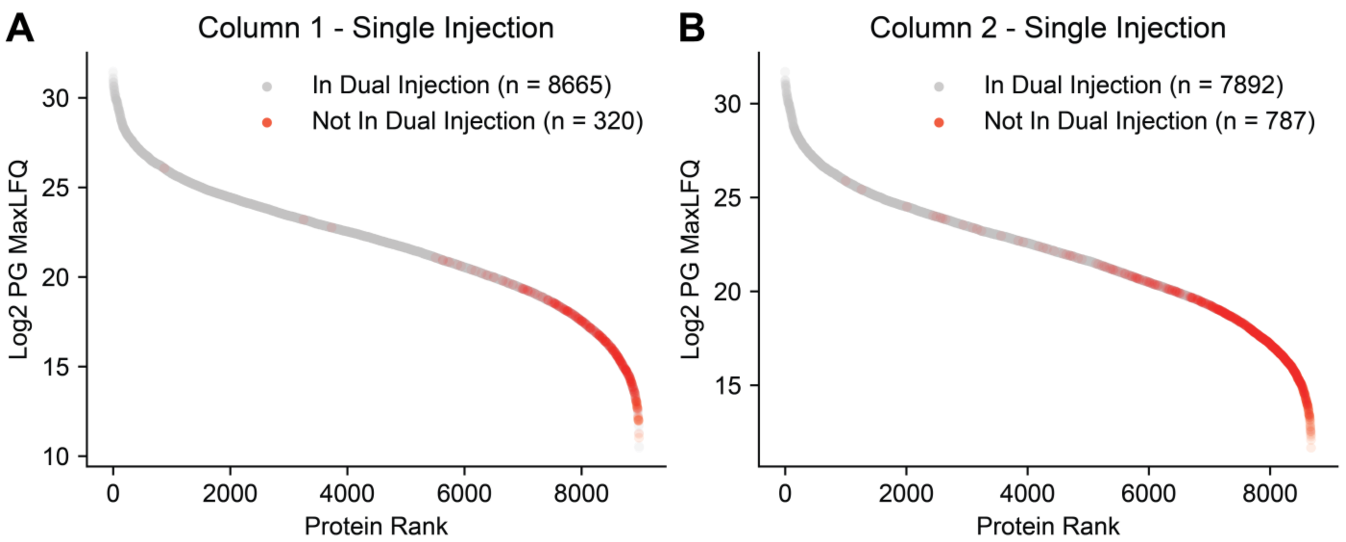

Figure S6: Dual vs. Single-Injection Dynamic Range. Protein group intensities for the single injections of mouse brain are plotted in ranked order for (A) Column 1 (corresponding to **Figure 2A**) and (B) Column 2 (corresponding to **Figure 2B**). Grey dots represent protein groups also detected for that column in the dual column injection (**Figure 2C**) and red dots represent proteins groups not detected in the dual column injection.

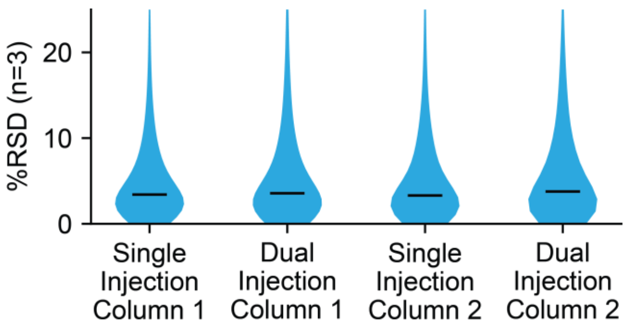

Figure S7: Protein Group RSD Zoom In. A zoomed in view of the protein group %RSD distributions shown in **Figure 3C** is shown. Note that this visualization cuts off the distribution at a %RSD of 25.

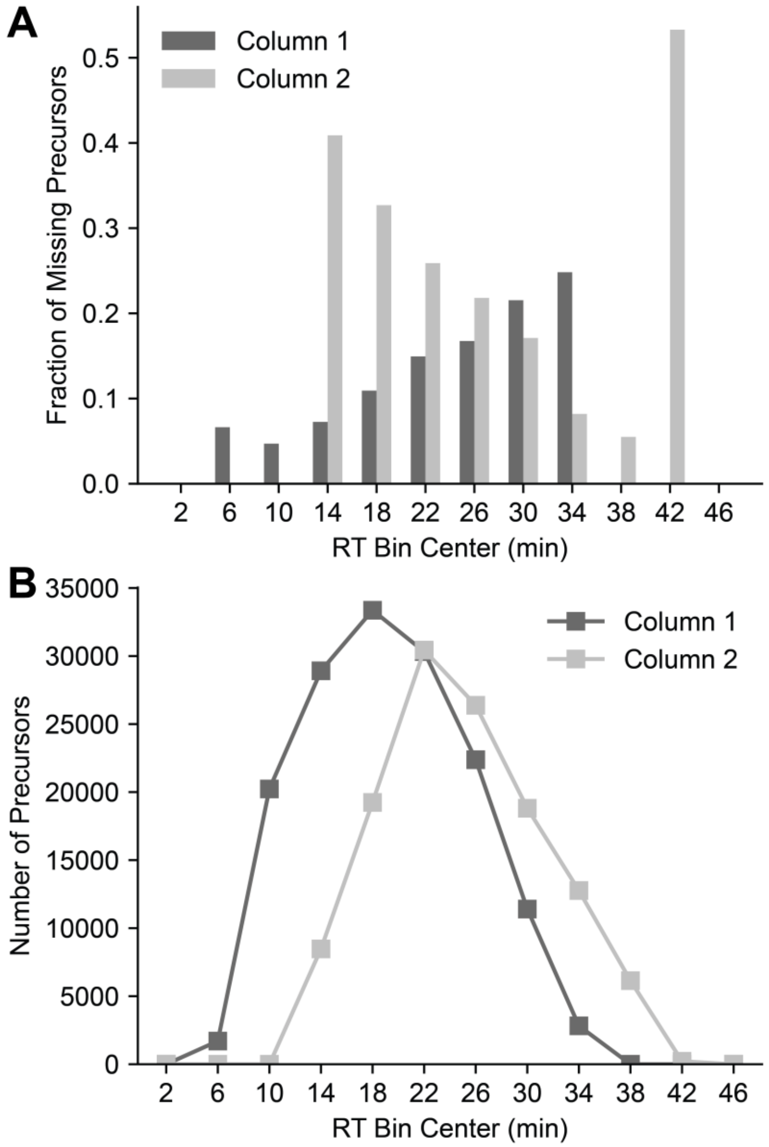

Figure S8: RT Binning Analysis of Dual Column SynchroSep-MS Data. (A) Precursors detected in the single-injection files for each column were binned by their detected retention time (RT). Then the fraction of these precursors that were missing in the dual-injection files was calculated and plotted as a function of the RT bin. (B) The number of precursors detected in the single-injection files were plotted as a function of the RT bin.

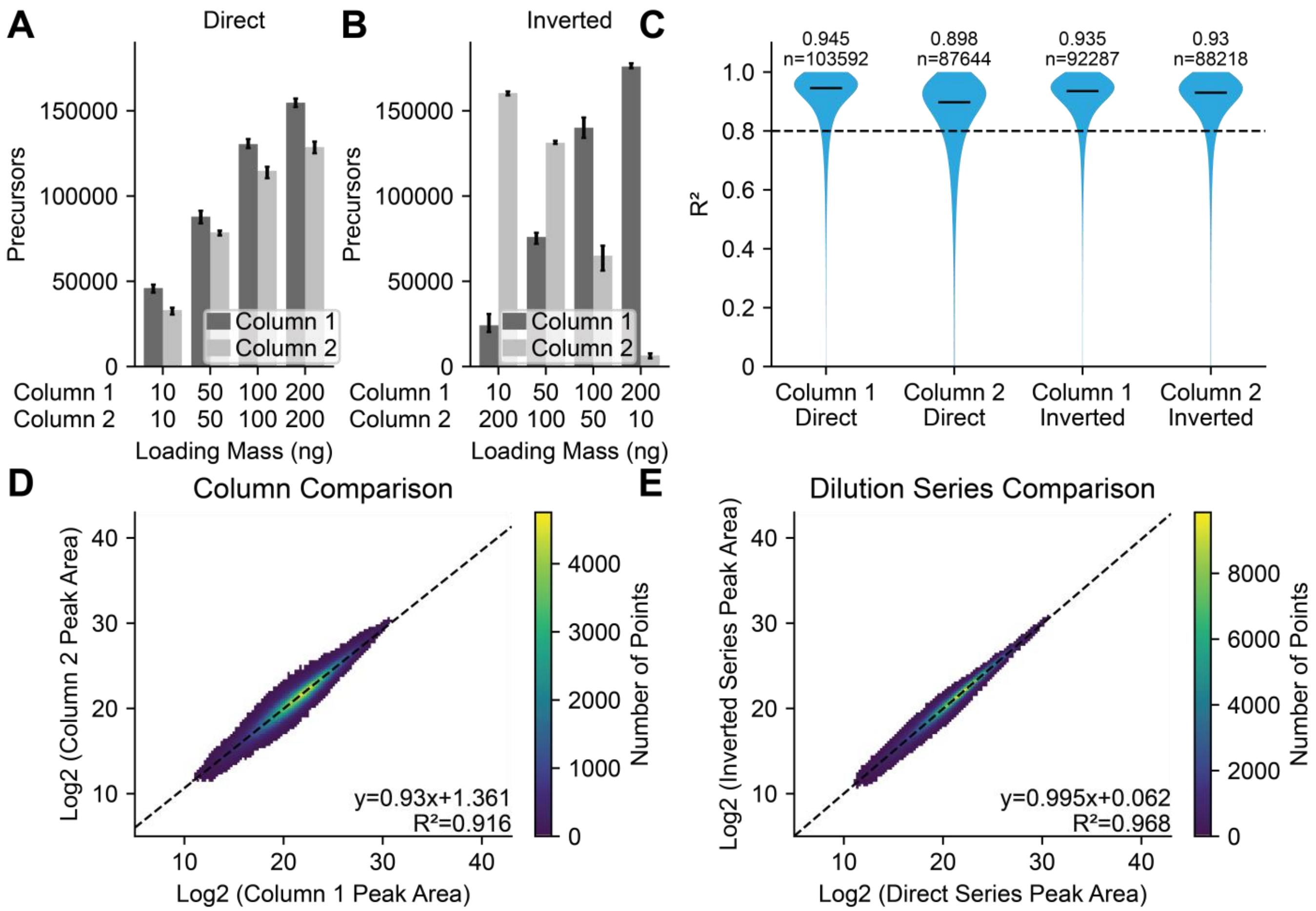

Figure S9. **Performance of SynchroSep-MS Method Assessed with Dilution Series – Precursor Level**. The number of precursors are shown for (A) the Direction Dilution Series and (B) the Inverted Dilution Series described in **Table 1**. The direct dilution series consists of injections of the same loading mass onto both columns, whereas the inverted dilution series uses different loads to represent an extreme case of varied background matrix from the other column. Error bars represent the minimum/maximum values observed across triplicate injections. (C) The R^2 value distributions for Column 1 and Column 2 across the dilution series shown in **Table 1** are shown. Values were only calculated for precursors detected across at least three concentration points. The median R^2 value and number of precursors included in each distribution are shown above each plot. The dashed line indicates an R^2 value of 0.8. (D) The log2-transformed peak areas for precursors detected across both Column 1 and Column 2 in the Direct Dilution series are plotted against each other. A slope less than 1 indicates that Column 1 peak areas are generally higher. (E) The log2-transformed peak areas for precursors detected across both dilution series are plotted against each other. A slope less than 1 indicates that the direct dilution series peak areas are generally higher. For this example, data on Column 1 is shown.

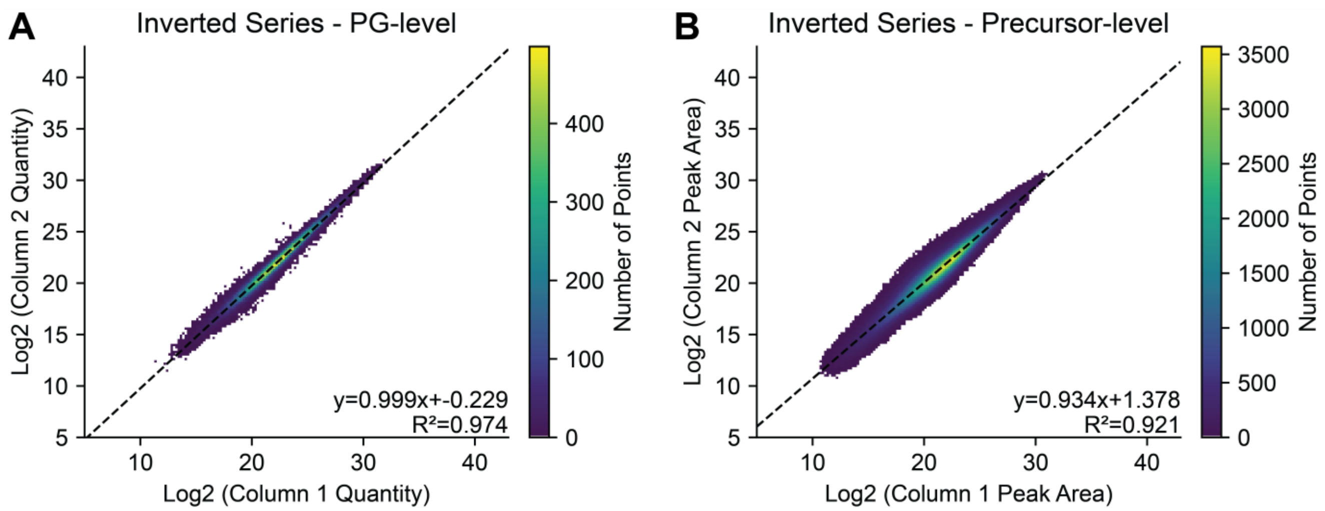

Figure S10: Column Intensity Comparisons for the Inverted Dilution Series. The log2-transformed quantities for (A) protein groups and (B) precursors detected across both Column 1 and Column 2 in the Direct Dilution series are plotted against each other. A slope less than 1 indicates that Column 1 protein quantities are generally higher.
